# Monounsaturated Fatty Acids in Obesity-Related Inflammation

**DOI:** 10.3390/ijms22010330

**Published:** 2020-12-30

**Authors:** Gaetan Ravaut, Alexandre Légiot, Karl-F. Bergeron, Catherine Mounier

**Affiliations:** CERMO-FC Research Center, Molecular Metabolism of Lipids Laboratory, Biological Sciences Department, University of Quebec in Montreal (UQAM), Montreal, QC H3C 3P8, Canada; ravaut.gaetan@courrier.uqam.ca (G.R.); legiot.alexandre@courrier.uqam.ca (A.L.); bergeron.karl-frederik@uqam.ca (K.-F.B.)

**Keywords:** monounsaturated fatty acids (MUFA), stearoyl-CoA desaturase-1 (SCD1), chronic inflammation, saturated fatty acid (SFA), metabolic syndrome

## Abstract

Obesity is an important aspect of the metabolic syndrome and is often associated with chronic inflammation. In this context, inflammation of organs participating in energy homeostasis (such as liver, adipose tissue, muscle and pancreas) leads to the recruitment and activation of macrophages, which secrete pro-inflammatory cytokines. Interleukin-1β secretion, sustained C-reactive protein plasma levels and activation of the NLRP3 inflammasome characterize this inflammation. The Stearoyl-CoA desaturase-1 (SCD1) enzyme is a central regulator of lipid metabolism and fat storage. This enzyme catalyzes the generation of monounsaturated fatty acids (MUFAs)—major components of triglycerides stored in lipid droplets—from saturated fatty acid (SFA) substrates. In this review, we describe the molecular effects of specific classes of fatty acids (saturated and unsaturated) to better understand the impact of different diets (Western versus Mediterranean) on inflammation in a metabolic context. Given the beneficial effects of a MUFA-rich Mediterranean diet, we also present the most recent data on the role of SCD1 activity in the modulation of SFA-induced chronic inflammation.

## 1. Inflammation in the Metabolic Syndrome

Obesity is the main factor responsible for the development of the metabolic syndrome, which is characterized by metabolic complications including visceral adiposity, hypertension, high circulating cholesterol and elevated glycemia [1,2,3]. This pathological combination often leads to insulin resistance and type 2 diabetes and is associated with a sustained inflammation profile [4,5]. In North America, people with a body mass index (BMI) superior to 30 are considered obese. This represents approximatively 36% of the population of North America and 13% worldwide [6].

Obesity is characterized by an excessive accumulation of lipids in adipose tissue. This accumulation becomes deleterious when it occurs in visceral fat [7]. In fact, waist circumference (as an indirect measure of visceral fat accumulation) is correlated with the development of specific metabolic disorders including cardiovascular diseases, hypercholesterolemia and type 2 diabetes [8]. When excessive lipid accumulation in adipose tissues occurs, ectopic accumulation (steatosis) appears in other tissues such as liver and muscle [8,9,10]. Saturated adipocytes release free fatty acids into the blood through the action of the Fatty acid translocase (FAT/CD36), the plasmatic Fatty acid binding protein (FABPpm) and the Fatty acid transport proteins (FATPs). These circulating free fatty acids are then captured by other organs, especially the liver and muscle, which gives rise to steatosis [11,12]. Accumulation of long chain fatty acids in non-adipose cells leads to the formation of toxic lipids such as ceramides and cholesterol esters [13]. These lipids induce lipotoxicity, leading to deleterious metabolic consequences including endoplasmic reticulum (ER) stress and inflammation [14,15].

Several population studies reveal that a low-grade and chronic inflammation is often developed in obese patients [16]. This is characterized by increased circulating levels of pro-inflammatory cytokines—especially Interleukin-6 (IL-6)—and of the chemokine MCP-1, both produced by the adipose tissue. Consequently, monocytes are recruited to the adipose tissue, inducing the secretion of other cytokines such as IL-1β and amplifying the inflammatory state [17,18]. In response to elevated cytokines levels, the liver secretes C-reactive protein (CRP), a key marker of inflammation associated with several metabolic diseases including type 2 diabetes and cardiovascular diseases [19,20,21,22]. CRP also aggravates disease development by activating the NF-κB signaling pathway, which is directly implicated in the expression of pro-inflammatory cytokines [23].

## 2. The Molecular Mechanisms of Inflammation

There are two main types of inflammation: acute and chronic. Acute inflammation appears in response to infections or injuries. This type of inflammation involves polynucleolar neutrophils and is characterized by apparition of swelling and heat around the damaged tissues. Activation of Toll-like receptors (TLRs) triggers the expression of inflammation effectors such as cytokines, prostaglandins, platelet activation factors, inflammasomes complexes, CRP, as well as NF-κB [24]. The resolution of this inflammation requires several conditions: destruction of the cause of inflammation, neutralization of pro-inflammatory markers (cytokines and prostaglandins) and clearance of neutrophils. These events typically occur in a few days, making this type of inflammation transient by nature [25].

The second type of inflammation, chronic inflammation, is sustained over time and is more deleterious for health. It often appears in individuals with poor feeding habits and a sedentary lifestyle, features strongly correlated with obesity development [26,27]. It is also present in different pathologies such as Alzheimer disease and asthma, and in several diseases associated with unbalanced metabolism such as atherosclerosis, cardiovascular diseases and type 2 diabetes [28,29,30,31]. Often named microinflammation or metabolic inflammation, it entails a complex mechanism involving crosstalk between various tissues (such as liver and adipose tissue) across the entire body. In general, this low-grade inflammation appears when cellular stress is recognized by the immune system [32]. Consequently, monocytes are recruited and infiltrate the tissues, becoming macrophages [24].

In inflammatory conditions such as obesity, two distinct macrophage subpopulations can be found in the affected organs. These are associated with different functions. The so-called M1 macrophages display an extreme pro-inflammatory state. They express high levels of pro-inflammatory receptors such as TLRs, Tumor necrosis factor receptors (TNFRs) and Interleukin-1 receptor (IL-1R), and exhibit a powerful activation of the NF-κB transcription factor necessary for the expression of pro-inflammatory cytokines. Conversely, the Μ2 macrophages are anti-inflammatory and are characterized by a higher expression of the Interleukin-4 receptor (IL-4R) whose activation downregulates inflammatory mediators such as TNF-α and IL-6. They also display an activation of the transcription factors PPARγ and PPARδ, which leads to higher expression of anti-inflammatory cytokines such as IL-10 [33]. The inflammation level present in tissues is therefore dependent on the balance between infiltrated M1 and M2 macrophages. This balance can be modulated by diet and hormonal status and is regulated by the PPARγ transcription factor [34].

A number of potential inflammation triggers have been identified in the context of chronic inflammation. TLR4 is activated by circulating long chain saturated fatty acids [35]. Consequently, the IKK-IκB signaling cascade leads to NF-κB nuclear translocation, where it activates the transcription of several pro-inflammatory cytokines and interleukins [36]. High circulating levels of pro-inflammatory cytokines such as TNF-α, MCP-1, TGF-β and IFN-γ, as well as of interleukins IL-6, IL-1β, IL-18, and IL-8, are observed in patients presenting an inflammatory state [37]. TLR4 activation is also linked to the increased expression of several proteins involved in the formation of inflammasomes, multiprotein complexes responsible for the activation of inflammatory responses. In particular for NLRP3 (NOD-like receptor family, pyrine domain containing 3), an inflammasome complex involved in several diseases associated with chronic and low-grade inflammation [38,39].

NLRP3 is considered an intracellular receptor responsible for the activation of inflammatory responses. Several factors can activate NLRP3 including elevated concentration of intracellular ATP, reactive oxygen species (ROS), mitochondrial oxidized DNA, and lysosomal destabilisation [40]. It can also be activated by low intracellular potassium or high calcium concentrations, which arise in response to cellular stress [40]. As NLRP3 is activated, the caspase 1 subunit of the NLRP3 complex cleaves pro-interleukins into mature IL-1β and IL-18, key circulating markers of low-grade inflammation [41]. NLRP3 is considered as a key factor responsible for the induction and progression of chronic inflammation. In fact, disruption of NLRP3 in adipose tissues decreases the concentration of pro-inflammatory cytokines and restores insulin sensitivity in obese mice [42].

**Figure 1:**
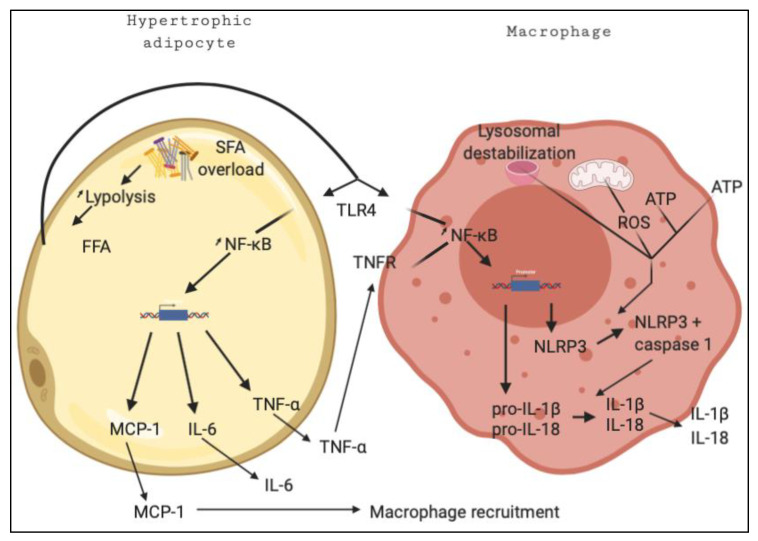
Crosstalk between adipocyte and macrophage leading to enhanced inflammation. FFAs (free fatty acids) produced as a consequence of SFA (saturated fatty acid) overload activate the TLR4 pathway, leading to MCP-1 (Monocyte chemoattracting protein-1), IL-6 (Interleukin-6) and TNF-α (Tumor necrosis factor alpha) secretion by adipocytes via NF-κB (Nuclear factor-kappa B) nuclear translocation. TNF-α activates TNFR (Tumor necrosis factor receptor) on recruited macrophages which, in combination with the TLR4 pathway, triggers NF-κB nuclear import and production of NLRP3 (NOD-like receptor family, pyrin domain containing 3), pro-IL-1β and pro-IL-18. Lysosomal disruption, as a consequence of ATP (adenosine triphosphate) and ROS (reactive oxygen species) accumulation, triggers NLRP3 activation and results in IL-1β/IL-18 maturation and secretion. This figure was generated with BioRender.

Another mechanism involved in the development of chronic inflammation involves excessive storage of triglyceride (TG) lipids within adipose tissues. Sedentary lifestyles and poor eating habits aggravate this unbalanced TG storage. In mice, excessive TG storage in white adipose tissue (WAT) induces secretion of pro-inflammatory adipokines such as IL-1β, TNF-α, MCP-1, and IL-6, triggering systemic metabolic inflammation [43]. In addition, excessive TG storage feeds lipolysis and increases the amount of intracellular and circulating free fatty acids (FFAs) (Figure 1). These fatty acids act as stress-inducing molecules which, captured by TLR4, induce activation of NF-κB and, in turn, induce NLRP3 expression in macrophages (Figure 1). In addition, intracellular FFAs can impair mitochondria and lysosome integrity, generating ROS (Figure 1) [44]. FFAs can also inactivate the serine-threonine kinase AMPK, an intracellular energy sensor. In this situation, secretion of IL-1β (via activation of the NLRP3 inflammasome) is increased and leads to lower insulin sensitivity [45]. Several authors even suggest that activation of AMPK can be considered as an anti-inflammatory marker in the context of metabolic inflammation [46,47].

## 3. Overview of Lipid Metabolism

Fatty acid molecules are structurally very diverse and, accordingly, are involved in several different biological functions. For example, phospholipids are an integral part of cell membranes while TGs are mainly involved in energy storage. There are two sources of lipids in the organism: dietary intake and de novo synthesis.

In humans, dietary lipids such as cholesterol, TGs, as well as long-chain saturated and unsaturated fatty acids are absorbed in the form of micelles by the intestinal enterocytes. Meanwhile, short and medium-chain fatty acids (2 to 10 carbon chain length) can directly cross enterocyte membranes and reach the bloodstream [48,49]. Enterocytes secrete lipids into the lymphatic and blood circulation in the form of chylomicrons. The liver then captures part of the chylomicrons, using the extracted lipids to assemble very low-density lipoproteins (VLDLs) containing Apolipoprotein B-100 (apoB-100). Secreted, circulating VLDLs transfer their lipids to the rest of the organism, becoming low-density lipoproteins (LDLs) in the process. In parallel to this system, enterocytes and hepatocytes secrete Apolipoprotein A-I (apoA-I) which, in complex with the uncaptured chylomicrons, forms high-density lipoproteins (HDLs) [50]. The main know function of HDLs is to sequester the cholesterol coming from peripheral organs and bring it to the liver [51].

Several mechanisms allow the intake of lipids into cells. Cholesterol is captured via the transmembrane Scavenger Receptor class B type I (SRB1) [52], while TG integrated into lipoproteins are hydrolyzed by the Lipoprotein lipase at the surface of epithelial cells. The FFAs generated are then absorbed by cells through different transporters such as the Fatty acid transport proteins (FATPs) and the Fatty acid translocase (FAT/CD36). The internalized FFAs are rapidly esterified into fatty acid-CoA, which can be then transformed back into TG. This esterification process involves various fatty acyltransferases such as GPAT (Glycerol-3-phosphate acyltransferase) and DGAT (Diacylglycerol O-acyltransferase). Newly formed TG are subsequently integrated into intracellular lipid droplets (LDs) where they are stored [53]. LDs are present in all eukaryotic cells. In normal conditions, lipids are preferentially stored into adipocytes, forming very large LDs. Under conditions where adipocytes are saturated (like obesity), lipids can be stored in other cells such as hepatocytes and myocytes, forming much smaller LDs [54]. This ectopic storage often leads to metabolic disorders and their associated inflammation.

**Figure 2:**
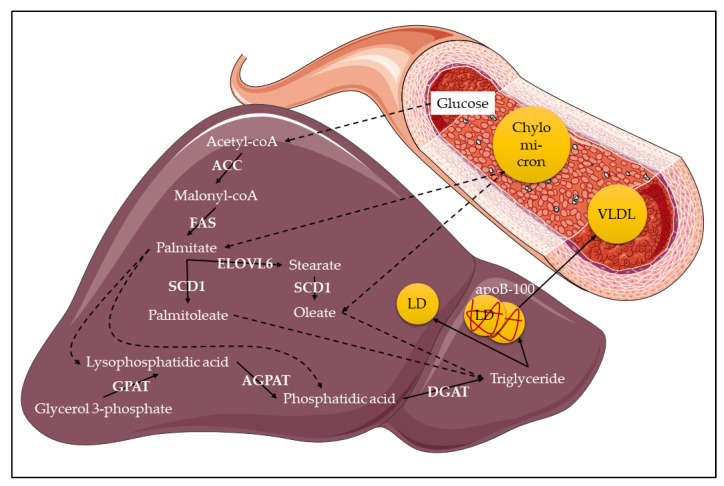
Triglyceride production in the liver. The chylomicrons bring fatty acids (mostly palmitate and oleate) to the liver, where they are used by GPAT (Glycerol-3-phosphate acyltransferase), AGPAT (1-Acylglycerol-3-phosphate-O-acyltransferase) and DGAT (Diacylglycerol-O-acyltransferase) enzymes to produce triglycerides. Alternatively, fatty acids can be synthesized de novo Figure 1. (Stearoyl-CoA desaturase-1) and ELOVL6 (Fatty acid elongase 6). Triglycerides are assembled into LDs (lipid droplets) and/or associated with apoB-100 (Apolipoprotein B-100) for secretion as VLDL (very low-density lipoproteins). This figure was generated with Servier Medical ART.

The other source of lipids in the organism comes from de novo lipid synthesis, also termed lipogenesis. This process occurs in most cells but, in humans, it principally occurs in hepatocytes (Figure 2) and adipocytes [55]. Lipogenesis synthesizes long-chain saturated fatty acids (palmitate) from acetyl-CoA generated by glucose hydrolysis. This synthesis is catalysed by the combined actions of Acetyl-CoA carboxylase (ACC) and Fatty acid synthase (FAS). Subsequently, saturated fatty acids (SFAs) are elongated by Fatty acid elongases (ELOVLs) [56] and/or desaturated by Stearoyl-CoA desaturases (SCDs), forming monounsaturated fatty acids (MUFAs) [57].

SCDs are the rate-limiting enzymes of MUFA formation. They are integrated into the ER membrane and are highly regulated by nutritional status and by hormonal regulators of appetite such as insulin [58,59]. SCDs introduce a delta-9 desaturation in SFAs stearate (C18:0) and palmitate (C16:0), forming the MUFAs oleate (C18:1n-9) and palmitoleate (C16:1n-7), respectively. These MUFAs are the main components of TGs (fatty acids that are preferentially stored) [60], cholesterol esters (cellular membrane components, precursors to steroid hormones and biliary acids) [61] and wax esters (compounds preventing evaporative water loss) [62]. They also constitute a large proportion of phospholipids comprising cellular membranes [57]. As such, SCDs are considered key regulators of lipid homeostasis, especially in liver and adipose tissue where lipogenesis is predominant. Modulation of SCD activity has been implicated in the development of the metabolic syndrome and its associated inflammatory state. Therefore, several studies have suggested targeting SCDs in order to treat various aspects of the metabolic syndrome, including type 2 diabetes and cardiovascular diseases [63,64,65].

In humans, there are two SCD isoforms, SCD1 and SCD5. SCD5 is mainly expressed in the brain, while SCD1 is more ubiquitously expressed [66,67]. In mice, the situation is more complex as four isoforms have been characterized (SCD1-4). They all share 85% amino acids homology with human SCD1, while SCD5 appears to be specific to primates. Mouse SCD1 is mainly expressed in lipogenic organs such as liver and adipose tissues. SCD2 is chiefly expressed in the brain, while SCD3 is found in the harderian, preputial and sebaceous glands. SCD4 expression has only be reported in the heart [68,69,70,71,72].

## 4. Stearoyl-CoA Desaturase-1

SCD1 is the most characterized SCD isoform. SCD1 transforms 85% of stearate and 51% of palmitate (from both dietary and lipogenesis origin) into MUFA [68]. Many studies have been performed in SCD1 knockout mice to better understand its role in metabolic processes. Global SCD1 knockout mice, in which every cell of the organism is SCD1 deficient, present with lack of sebum secretion and of lacrimal surfactant [73]. The lack of sebum gives rise to very dry skin with less hair and has led to the consideration of topical SCD1 inhibition as a potential treatment for acne.

Global SCD1 knockout mice are protected against obesity [74], insulin resistance [75] and fatty liver disease [61], as induced by both high-carbohydrate diet (HCD) [76] and high-fat diet (HFD) [74,75]. These mice display increased levels of plasma ketone bodies while the levels of circulating insulin and leptin are reduced [75]. Glycemia is also improved, as determined by a glucose tolerance test. The metabolic profiles of global knockout mice are more beneficial than their wildtype counterpart, as seen through the upregulation of lipid oxidation and the downregulation of lipid synthesis genes [74,76]. Because of the difference of global knockout mice, mice with a specific deletion of SCD1 in the liver are only protected from the deleterious effects of HCD (and not HFD). Under HCD, liver-specific knockout mice show a reduction of hepatic lipogenic enzyme gene expression as well as a reduction of plasmatic TG relative to controls [76]. As could be expected, these mice display a decrease of hepatic steatosis and associated metabolic complications such as hypercholesterolemia. This is consistent with diminished activation of SREBP-1 (as measured by protein maturation and nuclear localization levels) and with increased protein expression of the lipolysis transcription factor PPARα and the mitochondrial uptake acyl transporter Carnitine O-palmitoyl transferase 1 (CPT1) in the liver of global SCD1-deficient mice [77]. However, under HFD, liver-specific knockout mice develop hepatic steatosis and insulin resistance [78]. The steatotic effect of HFD on liver-specific knockout mice is probably due to the presence of SFA in the diet, which can be desaturated and integrated into TG, and, subsequently, into chylomicrons by enterocytes that still express SCD1. The chylomicrons can then be captured by the liver leading to hepatic steatosis and associated hepatic dysfunctions [76,79].

SCD1 expression is chiefly controlled by the lipogenic transcription factor SREBP-1c [77,80]. Under post-prandial conditions, the rise of lipemia and glycemia induce insulin secretion, one of the most important lipid anabolic hormones. Insulin activates the PI3K-PKB-mTORC1 signaling pathway, which induces the nuclear translocation of SREBP-1c and activates expression of enzymes involved in lipogenesis, including SCD1 [81]. There are others lipogenic transcription factors activated by dietary and hormonal factors such as insulin and glucose. Expression of lipogenic genes such as SCD1, FAS and ELOVL6 is triggered by the Liver X receptor (LXR), which is activated by insulin and by the Carbohydrate response element binding protein (ChREBP), itself activated by glucose [82]. One of the main LXR targets in lipid metabolism (especially of the LXRα isoform) is SREBP-1c, driving the expression of SCD1 [83]. Furthermore, MUFA (products of SCD activity) can regulate lipogenesis through AMPK phosphorylation [84,85]. Phosphorylated AMPK inhibits the mTORC1 complex [86], reducing the nuclear translocation of SREBP-1c and the expression of lipogenic genes like SCD1.

## 5. Role of Saturated Fatty Acids in Inflammation

### 5.1. Human Studies—Effect of Dietary SFAs

The type of lipids present in animal organisms is strongly influenced by diet [87]. Dietary SFAs are deleterious to metabolic health as they play an important role in the development of obesity, metabolic syndrome and chronic inflammation [88]. In fact, high SFA levels in the diet can be considered a pro-inflammatory factor in itself. Several studies have described clear correlations between the consumption of Western diets, rich in SFA, and the presence of obesity, hepatic steatosis and type 2 diabetes in humans [89,90,91]. Acute intake of SFA-rich diets triggers the development of an inflammatory profile in human subcutaneous adipose tissues, which includes increased expression of several genes involved in the synthesis of pro-inflammatory chemokines and cytokines [92]. In addition, compared to unsaturated fatty acid-rich diets, SFA-rich diets increase lipid storage within adipose tissues [90]. The adipocytes develop larger LDs and, therefore, contain more TGs. This increased intracellular TG pool leads to increased leptin secretion by adipocytes [93]. Furthermore, high circulating leptin is correlated with increased macrophage secretion of IL-1β, IL-6 and TNF-α [94,95]. A clinical trial has shown that a single 1000 kcal meal containing 60% fat (mainly SFA) leads to elevated plasmatic IL-6 concentrations [96]. This type of systemic inflammation is associated with vascular damages leading to coronary heart disease [96].

### 5.2. Animal Studies—Effect of Dietary SFAs

In agreement with observations made in humans, feeding rodents with diets rich in saturated fat increases hepatic and plasmatic TG levels and raises circulating IL-6 concentration [97,98]. Animals also develop glucose intolerance while macrophage recruitment in the liver is increased [97,99]. This suggests that inflammation is a consequence of diet-induced metabolic changes. Indeed, mice fed during 15 weeks with a HFD containing a majority of SFAs display an increased expression of hepatic TLR4 [98]. These animals also show elevated plasmatic concentration of IL-6, TNF-α and MCP-1, and lowered plasmatic concentration of the anti-inflammatory cytokine IL-10 [98].

Mice under SFA-rich HFD develop muscle steatosis due to accumulation of palmitate and stearate [100]. SFAs can also induce inflammation in the central nervous system. Brains of mice fed during 8 weeks with a HFD (composed mainly of SFA) display high concentrations of inflammatory markers (IL-6, IL-1β and TNF-α) and low levels of IL-10 [101]. Mice on a SFA-rich diet for as little as 4 weeks show elevated activation of NF-κB and, through TLR4 activation in the hypothalamus, expression of inflammatory markers (IL-1β, TNF-α and IFN-γ) in the brain as well as in the plasma [102,103]. This inflammation can even contribute to the development of obesity, at least in mice. Sustained HFD-induced inflammation in the arcuate nucleus, a specific region of the hypothalamus that regulates energy homeostasis, triggers microglia recruitment and fosters the death of satiety neurons [104].

### 5.3. Cellular Models—Effect of Exogenous SFAs

In vivo studies are realized with diets containing a mix of several types of fatty acids, which are at least partially transformed during the digestion process. This complicates interpretation of the results of these studies. Therefore, treatment of cultured cells with exogenous fatty acids has been used to determine the effect of specific SFAs expected to be found in post-prandial circulation.

Adipocyte cell models can provide insight into the in vivo mechanisms taking place within adipose tissue. Incubation of 3T3-L1 preadipocytes and rat primary epididymal adipocytes with palmitate for 24 h induces TNF-α and IL-6 secretion [105]. This treatment also increases the release of Monocyte chemoattractant protein-1 (MCP-1) [106,107], which has the potential to induce the recruitment of macrophages in vivo as well as their polarization into a M1 pro-inflammatory state. Exposition of pancreatic β cells (1.1B4 human cell line and rat primary cells) to palmitate increases secretion of IL-6 and IL-8, as well as ROS production. It is also associated with impaired insulin secretion [108,109]. This process has the potential to explain, at least in part, why saturated fat-rich diets lead to the development of type 2 diabetes.

In mouse microglia BV2 cells, palmitate treatment during 4 h induces IL-1β, IL-6 and TLR4 gene expression, as well as NF-κB induction [103]. In the RAW 264.7 mouse macrophage cell line, lauric acid (a 12-carbon chain SFA) can directly bind TLR4 and activate the nuclear translocation of NF-κB. This subsequently activates the expression of pro-inflammatory cytokines, especially TNF-α [110,111]. Treatment of RAW 264.7 cells with palmitate inhibits the expression of the transcription factor PGC-1β, which indirectly activates the nuclear translocation of NF-κB [112]. This leads to increased secretion of inflammatory cytokines TNF-α and IL-1β in the medium. Interestingly, when this medium is added to cultured 3T3-L1 preadipocytes, activation of the PI3K-PKB pathway is impaired, suggesting a decrease in insulin sensitivity [113].

The effect of SFAs on muscle cells has also been studied in vitro. Treatment of C2C12 mouse myotube cells with palmitate increases lipid storage as observed via lipid droplet size [114]. As for other cell types, this intracellular lipid accumulation causes lipotoxicity (elevated ROS and ER stress) and insulin resistance (disruption in PKP signaling). It also triggers NF-κB nuclear translocation, leading to the expression of pro-inflammatory cytokines such as TNF-α [114].

## 6. Role of Monounsaturated Fatty Acids in Inflammation

### 6.1. Human Studies—Effect of Dietary MUFAs

While SFAs increase inflammation, unsaturated fatty acids often have the opposite effect. Polyunsaturated fatty acids (PUFAs), especially the omega-3 class, have favorable effects on health. Several population studies have indeed demonstrated that, compared to SFA-rich Western diets, diets rich in omega-3 PUFAs exert beneficial metabolic effects at least in part by decreasing inflammation [115,116,117]. The effects of MUFAs on inflammation are less documented, but more and more evidences link MUFAs to anti-inflammatory states [92].

Dietary lipids are assimilated in the gut and then transported throughout the entire organism where they influence organ metabolism. Higher MUFA consumption increases MUFA levels, and reduces both SFA and PUFA, throughout the body [118]. The type of lipids present in our body can therefore be modulated through nutrition.

The impact of the Mediterranean diet has been studied in humans, including in several randomized crossover studies (Table 1) [119,120,121]. This diet is characterized by a high consumption of fish, olive oil, fruits and vegetables, and whole grains. In this type of diet, fat constitutes one third of the total kcal absorbed with almost 60% MUFA and 20% SFA [122]. For comparison, the Western diet has a similar amount of total fat but with a much lower proportion of MUFA (36% MUFA and 33% SFA) [119]. Compared to other diets, the Mediterranean diet is associated with lower blood pressure, as well as improved glucose and lipid blood profiles [123,124,125]. The Mediterranean diet lowers cardiovascular disease risk and even leads to beneficial gut microbiome changes: increasing *bacteroides*, *prevotella* and *faecalibacterium* genera, which are known to improve general metabolic health and prevent atherosclerosis and thrombosis (Table 1) [121,126]. In fact, olive oil, one of the main components of the Mediterranean diet, has been characterized as a prebiotic improving the host-microbial ecosystem (Table 1) [120].

Interestingly, supplementation of food with olive oil (an oil that is naturally enriched with the SCD1 product oleate) correlates with low occurrences of obesity and metabolic syndrome, and therefore, less chronic inflammation and mortality [127,128]. Furthermore, people consuming a Mediterranean diet generally show lower levels of the systemic inflammation profile that often appears when Western or carbohydrate-rich diets are consumed (Table 1) [129,130,131,132]. Consumption of Mediterranean diet for 3 to 4 weeks is also correlated with increased secretion of adiponectin, an adipokine with anti-inflammatory effects [94,133]. Similar observations on inflammation are made when subjects are fed with olive oil (Table 1) [131,134,135]. Subjects fed with a diet rich in olive oil for a period ranging from 3 weeks to 2 years display lower levels of circulating mononuclear cells (monocytic cells involved in the inflammatory response). In addition, their plasmatic pro-inflammatory cytokine levels (such as TNF-α, MCP-1, IFN-γ, CRP, IL-18, and IL-6) are lower when compared to subjects on a Western diet for the same period of time [131,136,137,138]. Compared to a one-time oral dose of a fat emulsion containing cow’s milk cream (25% oleate and 26% palmitate), an emulsion of olive oil (70% oleate and 15% palmitate) generates a more favorable lipid plasmatic profile, including a higher plasmatic concentration of MUFA-rich TG. Interestingly, in the same study, the authors incubated mouse BV2 microglia cells with purified plasmatic lipoproteins from these subjects. Upon treatment, the incubated cells switched from a M1 pro-inflammatory state to a M2 anti-inflammatory state in the presence of MUFA-rich TG (Table 1) [139]. This observation has been confirmed in another study on isolated human blood monocytes [140].

Anti-inflammatory effects of MUFA have been reported when MUFA are part of a dietary intervention. However, increased MUFA levels in vivo do not always have positive impacts on inflammation. In patients with chronic kidney disease, an elevated MUFA/SFA ratio in blood lipids—presumably reflecting increased SCD1 activity—is correlated with high levels of circulating CRP, suggesting an aggravation of inflammation [141]. In obese patients that underwent bariatric surgery, the concentration of lipids in SAT (subcutaneous adipose tissue) and VAT (visceral adipose tissue) was measured by gas chromatography. The MUFA proportion in these SAT and VAT samples is negatively correlated with inflammation and obesity-related conditions such as insulin resistance and type 2 diabetes, as measured by gene expression [91].

Though the beneficial effects of the Mediterranean diet cannot simply be attributed to its high olive oil content, as it also contains many omega-3 fatty acids, these population studies strongly suggest that dietary MUFA have anti-inflammatory effects, especially compared to SFA-rich diets such as the Western diet.

### 6.2. Animal Studies—Effect of Dietary MUFAs

To further investigate the effects of MUFA in the diet, several studies have been performed on HFD-fed mice. These animals allow for measurements of metabolic and inflammatory markers throughout the organism, rather than only in the blood or on surgical samples. Mice raised for 4 weeks on a diet rich in olive oil show elevated plasmatic concentration of MUFA with no change in hepatic SCD1 gene expression [142]. As in humans then, MUFA-rich diets can be used to study the effects of MUFA on systemic responses in rodents.

Studies performed in mice raised on a MUFA-rich diet for 15 weeks show higher circulating levels of anti-inflammatory markers (IL-4 and IL-10) and lower levels of pro-inflammatory markers (IL-6, MCP-1, IL-1β and TNF-α) compared to mice fed with a SFA-rich diet [98]. Even in obese and hypercholesterolemic mouse models, a MUFA-rich 8-week-long diet improves metabolic features, increasing the expression of anti-inflammatory markers such as IL-4, IL-10 and PPARγ. In addition, a decrease in circulating level of pro-inflammatory IL-6, MCP-1, TNF-α, and IL-1β, and a larger proportion of M2 macrophages (compared to M1) is observed in adipose tissues [143]. In a study performed in male Wistar rats, animals were raised 12 weeks on HFDs (35% kcal from fat) with different SFA/MUFA/PUFA ratios [144]. Compared to a diet containing a higher proportion of SFA, increasing the proportion of MUFA improves insulin sensitivity and induces expression of the anti-inflammatory cytokine adiponectin, especially in subcutaneous adipose tissue. However, MUFAs are less effective than PUFAs in inducing the expression of the adiponectin gene. Higher MUFA or PUFA proportions are also correlated with lower circulating LDL-cholesterol levels [145]. The effects of fatty acids on inflammation were studied in mice fed for 15 weeks with isocaloric diets rich in either SFA or MUFA [98]. Compared to the SFA group, liver analysis of mice fed with MUFA shows less macrophage infiltration as well as a decrease in TG content and lipid peroxidation (measured via thiobarbituric acid reactive substances). The plasmatic lipid profile is improved, as well as insulin sensitivity (as measured by HOMA-IR). The levels of circulating pro-inflammatory CRP and MCP-1 are also decreased [98]. Interestingly, a very recent study has shown that switching mice from a SFA-based HFD to a MUFA-based HFD partially attenuates the progression of hyperglycemia, diminishing pancreatic inflammation and ameliorating β cell function [146]. Macrophage infiltration in the pancreas was lower in MUFA-HFD fed mice. The authors suggest that this effect is mediated by AMPK [147]. Interestingly, when compared to a diet rich in n-6 PUFA, an olive oil-rich 24-month-long diet protects cardiac mitochondria from age-related damages in rats [148]. A very promising study has recently shown that, compared to SFAs, dietary MUFAs reduce the pro-inflammatory profile in the mouse brain (and in human blood), stimulating M2 macrophage polarization. The authors even propose to use olive oil in nutraceutical strategies to treat diseases associated with a neuro-inflammatory profile [139].

### 6.3. Cellular Models—Effect of Exogenous MUFAs

**Figure 3:**
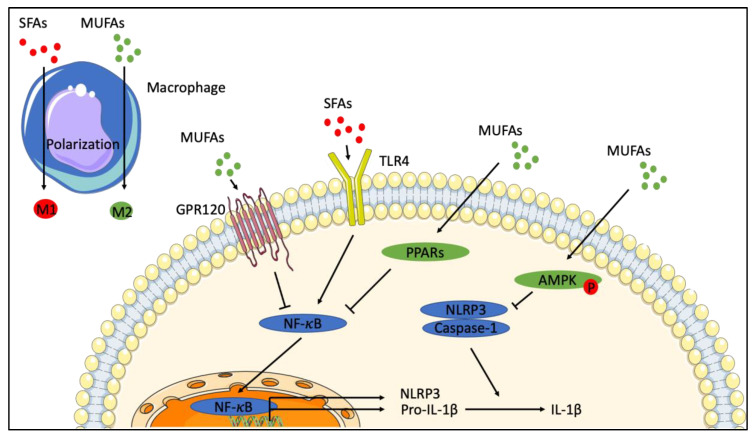
Monounsaturated fatty acids have anti-inflammatory effects. SFAs (saturated fatty acids) activate TLR4 (Toll-Like receptor 4) to induce NF-κB (Nuclear factor-kappaB) nuclear translocatable 3. (NOD-like receptor family, pyrin domain containing 3) and pro-IL-1β (pro-Interleukin-1beta) expression, leading to IL-1β secretion and macrophage M1 polarization. MUFAs (monounsaturated fatty acids) can inhibit NF-κB and NLRP3 activation, respectively, through direct binding to GPR120 (G-protein coupled receptor 120) or PPARs (Peroxysome proliferator activated receptors), and through AMPK (AMP-activated protein kinase) phosphorylation. By inhibiting macrophage M1 polarization, MUFAs potentiate M2 polarization. This figure was generated with Servier Medical ART.

Oleate protects HepG2 cells (a human hepato-carcinoma cell line) against SFA-induced lipotoxicity, decreasing ER stress, ROS production, and activation of inflammation markers (NLRP3, IL-6, MCP-1 and IL-1β) [149]. In primary murine hepatocytes, intracellular LD-derived MUFAs bind to SIRT1 (NAD-dependent protein deacylase sirtuin-1/silent information regulator 1), resulting in the activation of PPARα via PGC-1α. Oleate is also a direct PPARα agonist [150]. These mechanisms inhibit NF-κB activity (Figure 3) [151,152], explaining, at least in part, the resorption of hepatic inflammation by MUFAs.

In the 3T3-L1 murine preadipocyte cell line, oleate treatment increases expression of the adiponectin gene [153], probably through PPARγ activation [154,155]. Adiponectin induces IL-10 secretion and inhibits IL-6 and TNF-α secretion [153], which has the potential to reduce local inflammation in vivo. Adiponectin can also reduce peripheral inflammation by enhancing M2 macrophage polarization (Figure 3) [154,155,156,157,158,159,160,161].

Bone marrow-derived macrophages prepared from HFD-fed mice present a pro-inflammatory profile including macrophage M1 polarization and elevated secretion of IL-6 and TNF-α (Figure 3) [162]. The treatment of these macrophages with palmitoleate can switch the polarization of macrophages to M2 (Figure 3) [162]. Palmitoleate also activates AMPK, leading to a decrease of NF-κB nuclear translocation (Figure 3). This increases the expression of several anti-inflammatory factors such as MGL2, IL-10, TGFβ1, and MRC1 [162,163]. Incubation of mouse adipose stromal vascular fraction and bone marrow primary cultures with oleate inhibits LPS-induced IL-1β secretion [45,164]. In this situation, AMPK is activated, which in turn inhibits NLRP3 activation (responsible for IL-1β maturation) (Figure 3) [45,164]. Similar observations were reported on primary rat pancreatic islet cells [165].

MUFA also display protective effects in several other cell lines. For instance, oleate protects mouse muscle C2C12 cells from palmitate-induced insulin resistance and ER stress [166]. In mouse podocyte cells, derived from kidney epithelium, SFAs activate the cell death pathways associated with ER stress. This effect is reversed by oleate [167]. In the human endothelial EAHy926 cell line, palmitoleate decreases pro-inflammatory IL-6, IL-8 and MCP-1 secretion, and downregulates NF-κB (via PPARγ stimulation), as compared to palmitate [168].

## 7. The Role of Stearoyl-CoA Desaturase-1 in Inflammation

### 7.1. Human Correlation Studies

Given that SCD1 is the major enzyme involved in MUFA synthesis, several authors have hypothesized that an increase in expression and/or activity of SCD1 could be correlated with an improvement of patient inflammatory profile.

In a study performed on young adults [169], a clear correlation was observed between the rs2060792 (A/G) single nucleotide polymorphism (SNP) upstream of the SCD1 gene and the level of circulating SFAs palmitate and stearate. European women bearing the major allele present with higher palmitate and lower stearate concentrations. Interestingly, this SNP was positively associated with obesity and a higher level of the circulating pro-inflammatory factor CRP, especially in women. In a study analysing surgical samples from human visceral adipose tissue of obese individuals, an enrichment of histone methylation (H3K4me3) in the SCD1 and IL-6 promoters was correlated with increased BMI. This histone methylation enrichment pattern was associated with lower SCD1 expression and higher pro-inflammatory TNF-α and IL-6 expression [170].

However, in overweight adults, high palmitoleate plasma concentrations, reflecting high SCD1 activity, is correlated with the occurrence of inflammatory fatty liver disease [171]. This increased SCD1 activity could be due to a compensatory mechanism triggered by high circulating concentrations of its substrate palmitate [20,172].

The results obtained in these human studies have not always shown a strict correlation between SCD1 activity and inflammation. This suggests that the level of endogenous synthesis is not the only factor behind the modulation of inflammatory state by MUFA.

### 7.2. Animal Genetic Models

Both human and animal dietary studies clearly argue for a beneficial role of MUFA on the inflammatory status. Given that MUFAs are a product of SCD1 activity, the deletion of this enzyme should reduce the availability of MUFA (and increase SFA accumulation), leading to increased inflammation.

SCD1-deficient mice are a useful tool to study the effect of endogenous MUFA synthesis on lipid metabolism and inflammation processes. The asebia mouse model is deficient for SCD1 due to a naturally occurring genomic deletion. As in the SCD1 knockout mice, asebia animals display eye inflammation, a lack of sebaceous glands, and an absence of hair within scarred dermis [173,174]. In skin-specific SCD1 knockout mice, expression of pro-inflammatory genes IL-6, TNF-α and IL-1β is increased around hair follicles [175,176]. By inducing follicle cell death, this inflammation contributes to hair loss [177].

Like SCD1 knockout mice, asebia mice are protected from HFD-induced obesity, hepatic steatosis and glucose intolerance [178,179,180]. However, compared to wildtype mice, they exhibit a complex inflammatory profile including circulating pro-inflammatory markers such as IL-6 and IL-1β [181]. Adipose tissue-specific SCD1 knockout mice are protected against Western diet-induced obesity and fatty liver disease [74]. Their WAT displays a lower concentration of MCP-1 and TNF-α compared to WAT from wildtype mice, even when they are raised on HFD (60% kcal fat, mainly lard).

Enterocyte-specific SCD1 knockout mice display an increase in pro-inflammatory markers IL-6 and TLR4 within their colon and ileum [182]. Interestingly, these enterocyte-specific effects can be rescued by an oleate-rich diet [183]. Intriguingly, enterocyte-specific SCD1 knockout mice show diminished expression of the TLR4 receptor in the jejunum, suggesting a protection against inflammation [182].

Liver-specific SCD1 knockout mice display an increase in pro-inflammatory markers IL-1β and TNF-α within their liver [184]. These knockout mice models exhibit a reduction in the expression of lipogenic markers ACC, FAS and SREBP-1c. This potential for diminished palmitate synthesis could attenuate the inflammatory effects of SCD1 depletion.

### 7.3. Cellular Models

Several studies address the specific role of SCD1 in cellular models of inflammation. Silencing or inactivation of the SCD1 gene in the murine preadipocyte 3T3-L1 cell line exacerbates the effects of SFAs, increasing the expression of the pro-inflammatory markers TGF-β, IL-6 and MCP-1, and decreasing the anti-inflammatory IL-10 [185,186]. Similar results are observed in the EndoC-βH1 human pancreatic β cell line. Silencing SCD1 aggravates the lipotoxic effect of palmitate on inflammatory marker expression and, interestingly, oleate and palmitoleate treatments rescue these effects [187]. Incubating RAW 264.7 macrophages with a conditioned media obtained from primary adipocytes isolated from global SCD1 knockout mice decreases expression of both TNF-α and IL-1β pro-inflammatory cytokines [188]. SCD1 silencing in mouse primary macrophages renders the TLR4 receptor hypersensitive, which exacerbates the gene expression of pro-inflammatory cytokine (IL-1β, MCP-1 and IL-6) [189]. TLR4 hypersensitivity is thought to stem from increased SFA proportions within membrane phospholipids [189].

Other technical approaches allow for insight into the effect of SCD1 overexpression. In primary human myotube cells, overexpression of SCD1 prevents palmitate-induced ER stress and IL-8 gene expression [190]. Mesenchymal stromal cells (MSC) can be prepared from posterior iliac crest bone marrow extracted from patients [191]. When these MSC cells are treated with T0901317 (an LXR agonist), SCD1 and LXRα expression are increased. This treatment reduces palmitate-induced Caspase 3/7 activation and expression of pro-inflammatory IL-6 and IL-8. When MSC cells are incubated with the specific SCD1 inhibitor CAY 10566, the effect of the LXR agonist is abrogated. This suggests that, at least in bone marrow stromal cells from these patients, SCD1 is involved in the prevention of inflammation and apoptosis induced by palmitate [191].

More recently, a study has been performed using primary hepatic cell isolated from G-protein coupled receptor 120 (GPR120) deficient mice. This receptor interacts with MUFA, especially palmitoleate [192]. The activation of GPR120 by palmitoleate, is involved in the resolution of palmitate-induced inflammation through a reduction of NF-κB activity. Interestingly, in these cells, a correlation between SCD1 expression and GPR120 activity is observed [193].

Inhibiting SCD1 in cells leads to increased inflammation. This is probably due to a combination of lower intracellular MUFA concentration and, undoubtedly, higher intracellular SFA concentration.

## 8. Conclusions

As presented throughout this text, dietary fat intake has an undeniable impact on inflammation. There is evidence that chronic low-grade inflammation can be prevented by lifestyle interventions. The SFA-rich Western diet can induce chronic inflammation and increase the risk of developing obesity-related metabolic disorders such as cardiovascular diseases, type 2 diabetes, and hepatic steatosis. At the opposite, a Mediterranean diet especially rich in oleate is favorable to an anti-inflammatory state and is associated with a decreased risk of metabolic syndrome development. Indeed, both human and animal diet studies have shown that substitution of SFA by MUFA activates beneficial anti-inflammatory mechanisms (M2 macrophage polarization, adipocyte IL-10 secretion, inhibition of NLRP3 inflammasome) and reverses the deleterious effect of SFAs on adipose tissues, hepatic tissue and β cells. Many mechanisms presented here can account for the protective effects of dietary oleate and high levels of circulating MUFAs. The addition of MUFA in diets can therefore be a potential nutraceutical avenue to decrease chronic inflammation and, subsequently, to ameliorate the general metabolic profile. In accordance with the beneficial effects of dietary MUFAs, some studies have shown that inhibiting SCD1 aggravated the deleterious effects of SFAs. This is probably due to an increase of SFA levels (SCD1 substrates). Thus, SCD1 is an interesting therapeutic target to decrease intracellular SFA concentration in favour of MUFA. However, other studies have shown that SCD1 inhibition can have favourable outcomes. SCD1 deletion protects mice against the deleterious effects of SFA-rich HFD and even improves the metabolic profile of humans and animals. In this case, the protective effects of SCD1 deletion cannot be attributed to MUFA activity in the organism. In fact, we and others have shown that SCD1 deletion inhibits lipogenesis [74,76,77,79,182]. This can be attributed to inhibition of SREBP-1c oleylation, decreasing its transcriptional activity [77]. This aspect of SCD1 activity deserves to be further investigated to better understand its specific role in inflammation.

## Figures and Tables

**Table 1:** Main outcome of human clinical trials involving MUFA-rich diets.

Studies	Participants	Duration	Population	Diet	Main Outcome
Martin-Pelaez 2017 [120]	12 volunteers, aged 46–67 years	3 weeks	Hypercholesterolemic volunteers	25 mg/day of virgin olive oil	Improved cholesterolemia profile
De Filippis 2016 [126]	153 individuals	1 week	Healthy people, omnivore/vegetarian or vegan	Mediterranean diet	Increase of *Prevotella* and *Firmicutes* within gut microbiota, improvement of atherosclerosis profile
Cesari 2018 [128]	421 subjects, aged > 90 years	12 months	NA	Mediterranean diet	Decreased risk factors for cardiovascular disease development
Schwingshackl 2015 [131]	3106 participants	>4 weeks	NA	Olive oil interventions	Improvement of inflammatory profile, CRP plasma level decreased
Paniagua 2007 [133]	11 volunteers	4 weeks	Diabetic subject	Mediterranean diet	Improved insulin sensitivity and adiponectin secretion
Konstandinidou 2013 [136]	90 participants, aged 20–50 years	1 year	Healthy volunteers	Mediterranean diet	Protective gene expression associated with inflammation improvement
Esposito 2004 [138]	90 patients	3 years	Diabetic patient	Mediterranean diet	Decreased insulin resistance, reduced plasma CRP and IL-6 level
Toscano 2020 [139]	6 volunteers, aged 25–35 years	NA	Healthy volunteers	One oral emulsion of olive oil	Enhanced M2 macrophage polarization, reduced proinflammatory profile

Abbreviations: NA (not applicable), CRP (C-reactive protein), IL-6 (Interleukin-6).

## Abbreviations

ACC	Acetyl-CoA carboxylase
AGPAT	Acylglycerol-3-phosphate-O-acyltransferase
AMPK	AMP-activated protein kinase
apoA-I	Apolipoprotein A-I
apoB-100	Apolipoprotein B-100
ATP	Adenosine Triphosphate
BMI	Body mass index
ChREBP	Carbohydrate-responsive element-bonding protein
CPT-1	Carnitine palmitoyltrasnferase-1
CRP	C-reactive protein
DGAT	Diglyceride acyltransferase
DNA	Desoxyribonucleic acid
ELOVL	Elongation of very long chain fatty acid
ER	Endoplasmic reticulum
FABP	Fatty acid binding protein
FAS	Fatty acid synthase
FAT/CD36	Fatty acid translocase/cluster of differentiation 36
FATP	Fatty acid transport protein
FFA	Free fatty acid
GPAT	Glycerol-3-phosphate acyltransferase
GPR120	G-protein-coupled receptor 120
HCD	High carbohydrate diet
HDL	High density lipoprotein
HFD	High fat diet
hMSC	Human mesenchymal stromal cells
HOMA-IR	Homeostatic model assessment of insulin resistance
IFN-γ	Interferon-gamma
IKK-IκB	IκB kinase-inhibitor of nuclear factor kappaB
IL-1β	Interleukin-1 beta
IL-10	Interleukin-10
IL-18	Interleukin-18
IL-1R	Interleukin-1 receptor
IL-4R	Interleukin-4 receptor
IL-6	Interleukin-6
IL-8	Interleukin-8
LD	Lipid droplet
LDL	Low density lipoprotein
LPS	Lipopolysaccharide
LXR	Liver X receptor
MCP-1	Monocyte chemoattractant protein-1
MGL2	Macrophage galactose N-acetyl-galactosamine specific lectin 2
MRC1	Macrophage mannose receptor 1 precursor
mTORC1	Mammalian target of rapamycin complex 1
MUFA	Monounsaturated fatty acids
NF-κB	Nuclear factor-kappaB
NLRP3	NOD-like receptor family, pyrin domain
PGC-1β	Peroxysome proliferator-activated receptor 1-beta
PI3K	Phosphoinisitide-3-kinase
PKB	Protein kinase B
PPARα	Peroxysome proliferator-activated receptor alpha
PPARδ	Peroxysome proliferator-activated receptor delta
PPARγ	Peroxysome proliferator-activated receptor gamma
PUFA	Polyunsaturated fatty acid
ROS	Reactive oxygen species
SAT	Subcutaneous adipose tissue
SCD	Stearoyl-CoA desaturase
SFA	Saturated fatty acid
SNP	Single nucleotide polymorphism
SRB1	Scavenger receptor class B type 1
SREBP-1	Sterol regulatory element-binding protein-1
TG	Triglycerides
TGF-β	Transforming growth factor-beta
TLR	Toll-Like receptor
TNF-α	Tumor necrosis factor-alpha
TNFR	Tumor necrosis factor receptor
VAT	Visceral adipose tissue
VLDL	Very low-density lipoprotein
WAT	White adipose tissue
